# Exploring the perceived restorativeness of natural soundscapes under the global pandemic of COVID-19: A moderated mediation model

**DOI:** 10.1371/journal.pone.0256855

**Published:** 2021-08-27

**Authors:** Mengyuan Qiu, Jie Zhang

**Affiliations:** 1 College of Economics and Management, Nanjing Forestry University, Nanjing, Jiangsu, PR China; 2 School of Geography and Ocean Science, Nanjing University, Nanjing, Jiangsu, PR China; Universidad de Malaga, SPAIN

## Abstract

Although emerging studies have discussed the potential benefits of soundscape in mental restoration, few have investigated how soundscape renews and re-energizes people, especially in facing the current public challenge of the COVID-19 crisis. We established a moderated mediation model to examine the relationship of the four restorative components of soundscape: being away, compatibility, extent and fascination. The data were collected in Xixi Wetland National Park, China, before the outbreak of COVID-19 (n = 562) in October 2019 and post COVID-19 in October 2020 (n = 341). The results revealed that natural soundscapes have great restorative benefits for visitors. The inter relationships of the restorative components are moderated by the perceived stress level which show significant different before and after the COVID-19 pandemic. The post-COVID-19 visitors reported a higher level of stress while natural soundscapes had greater effects on their mental restoration. The direct effects of extent and fascination as well as the mediating effects of fascination were stronger among the post-COVID-19 group. However, the path coefficient from being away to compatibility were higher in the pre-COVID-19 group. This study improves the current understanding of the interactive mechanism among the restorative components of soundscape. Knowledge about natural soundscape encourages practitioners to consider it as a guideline for the creation of sustainable environments, especially under the COVID-19 crisis.

## Introduction

As of April 19, 2021, 3,034,222 people have died from the COVID-19 outbreak. There are currently 142,059,494 confirmed cases in 219 countries and territories. Unfortunately, the coronavirus death toll is still rising. According to the data compiled by WHO, COVID-19 has seriously disturbed people’s normal lives worldwide. Stringent control measures have been enforced in over 90 countries, such as tourism ban, school closures, meeting cancelations, and so on [1]. The isolation policy caused negative effects not only on economic development, but also on public health and well-being. Based on the Attention Restoration Theory (ART), restoration is the renewal of “adaptive resources” depleted in meeting the demands of everyday life [2]. In the near feature, the public’s rapidly increasing need of mental restoration will raise a global concern [3].

Soundscape can be described as “the sonic environment, which is perceived and understood by an individual or the society in context” [4]. A growing number of people wonder when they will be able to get close to nature to heal their bodies and minds since they are under strict lockdown in their homes [2, 5]. Research has suggested that listening to natural sounds releases stress and increases happiness [6]. For example, despite the invisibility of birds in the forests, their crisp songs help visitors relax [7].

Payne described that being away, compatibility, extent and fascination are the critical components for natural soundscapes in producing restorative effects and developed the Perceived Restorativeness Soundscape Scale (PRSS) to assess the perceived restorativeness of natural soundscapes [8]. Even though various studies have revealed that natural soundscapes have great potential on mental restoration, most of them failed to further dissect the restorative components of the natural soundscapes, such as, which restorative components of natural soundscapes most effectively promoted attention recuperation, and how these restorative components of natural soundscapes relate to fatigue recovery. Moreover, the studies did not delve into what makes natural soundscapes re-energize people [9]. Especially, the outbreak of COVID-19 has caused new stressors and trauma for the mental health of the public. There is little knowledge about how people restore their mental health from natural soundscapes during the post-COVID-19 period.

To fill the gap existed in soundscape and restoration research, the objective of this study is to explore how the four components of natural soundscapes interplay to facilitate attention restoration and what’s the difference of the perceived restorativeness soundscape under the global pandemic of COVID-19. The comparative study between the pre- and post-COVID-19 visitors in Xixi National Wetland Park allows us to determine if the restorative process is consistent or has some changes in the new stressful context [10]. By developing important insight about the perceived restorativeness soundscape across different periods of visitors, this study will contribute to the advancement of knowledge to unveil the components of natural soundscape beneficial for visitors’ mental restoration. A clear understanding of the relation among the components of natural soundscapes and mental health will benefit Destination Management Operators (DMOs) and policy makers to pay attention to auditory cues to reduce stress, depression and other mental disorders during and after the COVID-19 pandemic.

## Literature review

### The restorative components of soundscape

The Perceived Restorativeness Soundscape Scale (PRSS) was designed to assess the potential of a natural soundscape in producing restorative perception [11, 12]. The PRSS was composed of four constructs: (1) Being away refers to the perception of natural soundscape alleviating attentional fatigue when the person takes from the stressful situation and moves to a different environment. (2) Compatibility refers to the mutual responsiveness of the natural soundscapes and people’s behavior which is in accordance with the sonic environment’s demands. (3) Extent is the scope and coherence of soundscape which has an explorative potential. If a sonic environment has insufficient scope and coherence, it is difficult to experience it as a unified entity. (4) Fascination is an alternative description for effortless, undivided attention to the natural soundscapes. A natural soundscape that has all these four components can be described as a restorative soundscape [13]. Overall, the higher the PRSS score, the greater the restorative potential of a natural soundscape.

A series of research has indicated that the four components of natural soundscapes provide attention restoration to various people. As Payne suggested, for the urban population, the rural soundscape was perceived as the most restorativeness since it is far away from their daily lives [9]. The study of Qiu et al. conducted an on-site research for 563 visitors in a Jiuzhai Valley National Park and found that the natural soundscapes are rich for exploration and thus allow visitors to relax and renew [14]. Listening to birdsongs arouses children’s involuntary attention and restores their direct attention, which is important for people to improve their psychological health [15, 16]. This is in line with the findings of Zhang et al., who conducted an evaluation of various soundscapes among highly stressed adults and found that the component scores of birdsongs were the highest, while urban noise has less restorative potential [17].

### The relationship among the restorative components

The restorative components of a natural soundscape (being away, compatibility, extent, and fascination) are interrelated and interactive with each other [18]. As Laumann stated, extent and fascination refer to the environmental features, as well as being away and compatibility relate to an individual’s perception. Therefore, the restorative components are in different hierarchies [19]. Another study compared the restorative components of urban and natural environments and found that the correlations among these components are significant in both settings. The causal relationship between extent and fascination is most notable in the natural environment [20]. However, empirical attempts to measure the restorative components of a natural soundscape and explore their relationships were not common in most general studies reviewed earlier.

According to the research that focused on the total environment, we assumed that the four components of PRSS are interrelated. Extent is a critical antecedent for fascination. It helps run a cognitive map of natural soundscapes so that theses soundscapes follow each other in a relatively sensible, predictable, and orderly way and demand less directed attention [21]. Therefore, the natural soundscapes could capture one’s attention in an automatic, bottom-up way and allow people to define the meaning of the soundscapes with respect to their intrinsic demands [22]. Subsequently, a physical or psychological being-away is induced by various *extent* and *fascinate* elements of the natural soundscape [23]. People would feel they are in a different setting than usual, and would be able to escape from unwanted distractions of their daily obligations. Finally, the perception of being-away from the ordinary changes the mind-set, making people aware of the positive relationship between an individual’s purpose or inclinations and the kind of activities supported, encouraged, or demanded by a natural soundscape [24]. *Extent* and *fascination* are also associated with *compatibility*, as the larger the scope and the more fascinating the natural soundscape appears, the more the compatibility between self and the natural soundscape [19]. Lehto separated being away into “physical away” and “mental away” which explain 37% of compatibility [25]. Therefore, the differences between these two components need to be resolved, providing opportunities for attention restoration.

To sum up, we hypothesize that the restorative components of natural soundscape have potential correlations. More specifically, we hypothesize as follows:

**Hypothesis 1 (H1):** The restorative components of natural soundscape are related.
  H1a: Extent directly influences fascination.
  H1b: Extent directly influences being away.
  H1c: Extent directly influences compatibility.
  H1d: Fascination directly influences being away.
  H1f: Fascination directly influences compatibility.
  H1g: Being away directly influences compatibility.
**Hypothesis 2 (H2):** Fascination of natural soundscape is a mediator in the model.
  H2a: Fascination plays a mediating role between extent and being away.
  H2b: Fascination plays a mediating role between extent and compatibility.
**Hypothesis 3 (H3):** Being away of natural soundscapes is a mediator in the model.
  H3a: Being away plays a mediating role between extent and compatibility.
  H3b: Being away plays a mediating role between fascination and compatibility.

### The influence of stress level on restorative soundscape

The perceived restorativeness of a natural soundscape depends on the level of each restorative component and its association with various variables (e.g., preference, complexity, context, culture) [4]. Specially, the perceived stress level has significant effects on their mental restoration [26]. The visitors with high stress levels are more sensitive to natural soundscapes than ordinary people [27]. When exposed to a tranquility area, the scores of the PRSS are higher for the most stressed individuals, and the soundscapes thus greatly improve their health and well-being [28]. Some studies have been criticized for not including a control group of low-fatigued individuals to compare with the highly-stressed people [29]. Considering this paradigm, it becomes impossible to determine whether the superior performance after listening to natural soundscapes signals a recovery from attention depletion [30]. A previous study during the outbreak of SARS found that the mental restoration of patients had different protective effects depending on their perceived level of stress [31].

The COVID-19 crisis created new stressors for public mental health. However, empirical studies of the restorativeness of natural soundscape in post COVID-19 are rather limited, and the effects of each restorative component under different levels of stress need to be investigated. This study hypothesizes that the perceived stress level is a moderator factor and thus the pre- and post-COVID-19 visitors can be regarded as two groups with different stress level. Therefore, the restorative components of natural soundscape have different inter-relationships due to the psychological stress caused by the COVID-19 pandemic.

**Hypothesis 4 (H4):** Perceived stress level is a moderator for the relationships of the restorative components.

**Fig 1:**
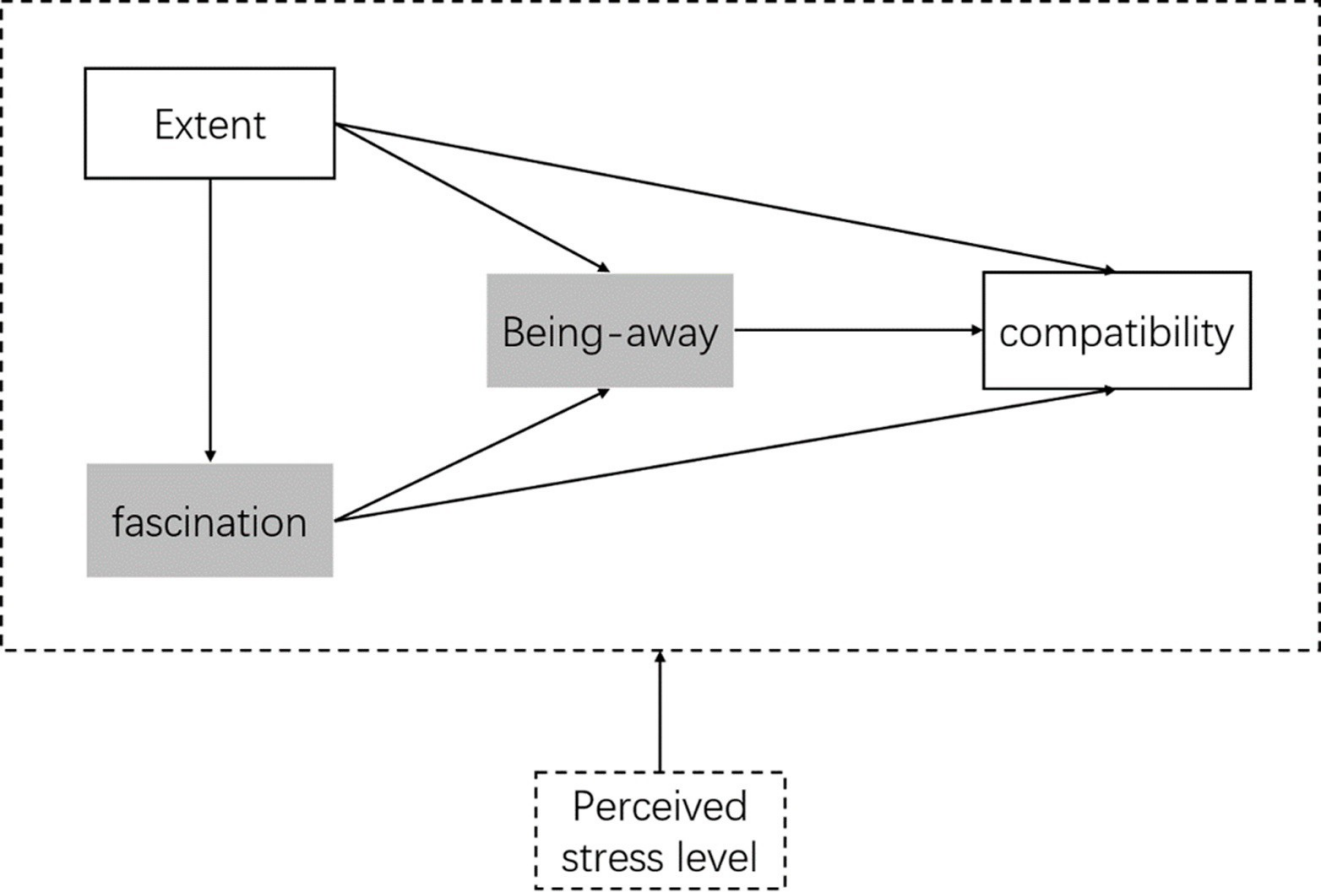
Hypothesized relationships among restorative components of natural soundscape.

According to all the hypothesis, we established a conceptual to explore relationships of restorative components for natural soundscapes (Fig 1).

## Methodology

### Measurements

We applied the Perceived Stress Scale-10 items (PSS-10) to evaluate the visitors’ mental state before they experienced natural soundscapes in Xixi National Wetland Park [32]. The PSS-10 was developed by Mitchell and translated into Chinese by the World Health Organization. The Chinese version has been used across different contexts after its reliability and validity were examined. This study employed the Chinese version of PSS-10 to describe the state of the participants’ perceived stress level before they visited the Xixi National Wetland Park. Responses were quantified on a 5-point Likert-type scale according to the frequency with which the stress situation occurred in the past month, with 1 = "never" and 5 = "very often”.

The restorative components of the natural soundscapes were the main variables. According to the PRSS developed by Payne, it comprised four constructs: being away, compatibility, extent, and fascination [9]. Laumann et al. split *being away* into two components: a physical being away (referred to as novelty) and a psychological being away (referred to as escape) [19]. This finding is plausible since the definition of being away contains a physical being-away from an ordinary environment and a physiological being away from unwanted distractions [2]. We used a second-order construct with three items each to measure being away. The other constructs referred to the self-reported scape originated by Payne. Some items were modified to fit the Chinese context. For example, “All the sounds I am hearing are harmony between man and nature” embodies the essence of the Taoist culture. Finally, 16 items were evaluated for the perceived restorativeness of the natural soundscapes. Responses were quantified on a 5-point Likert-type scale, with 1 = “Do not feel” to 5 = “Fell very strongly”.

Therefore, the questionnaire used in study contain three parts, the Perceived Stress Scale, the Perceived Restorativeness Soundscape Scale and the demographic characteristics of participants. A pilot test aimed to examine the content and face validity of the questionnaire was conducted from June 8 to 10, 2019. There are 50 university students who have been visited to Xixi National Wetland Park was selected by convenient sampling. The KMO value and the Cronbach’s alpha coefficient exceeds 0.85 and 0.90, respectively. It had shown fairly good reliability and validity of the self-reported scale. The questionnaire could be used for formal survey.

### Study site

**Fig 2:**
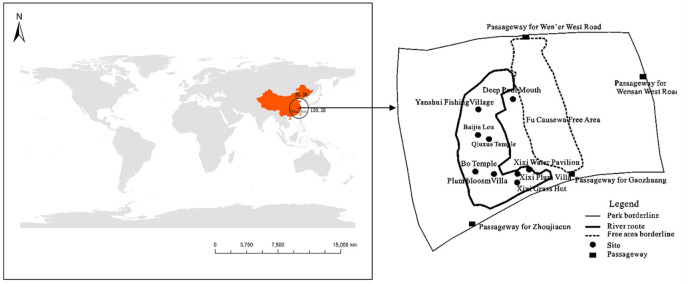
Geographical location of Xixi National Wetland Park.

The Xixi National Wetland Park, which is full of natural soundscapes, was an appropriate location for the current study. It is located in the northwest of Hangzhou, Zhejiang Province, which is 188 kilometers away from Shanghai. As China’s first and only national wetland park, Xixi National Wetland Park combines urban life, farming, and wetlands and covers an area of 10 square kilometers (Fig 2). There are 769 types of vascular plants, 867 species of insects, and 195 species of birds in Xixi National Wetland Park, providing an ideal environment with abundant sights and sounds of flora and fauna. Moreover, water accounts for 70% of the area, providing plentiful supply of geographical and meteorological sounds in ponds, lakes, and swamps. In 2019, Xixi National Wetland Park received 18.9542 million visitors. Despite the impact of the Covid-19 pandemic, the number of visitors to Xixi National Wetland Park on the National Day of 2020 was 10,700. The sound album “Listen to the Xixi wetland—the sound of nature” was launched in Himalaya, the largest music sharing platform in China. The natural soundscapes of Xixi National Park were so popular that the total number of listeners topped 80 million until Dec. 2020. This made the natural soundscapes of Xixi one of the most well-known attractions for visitors.

### Participants and procedures

**Fig 3:**
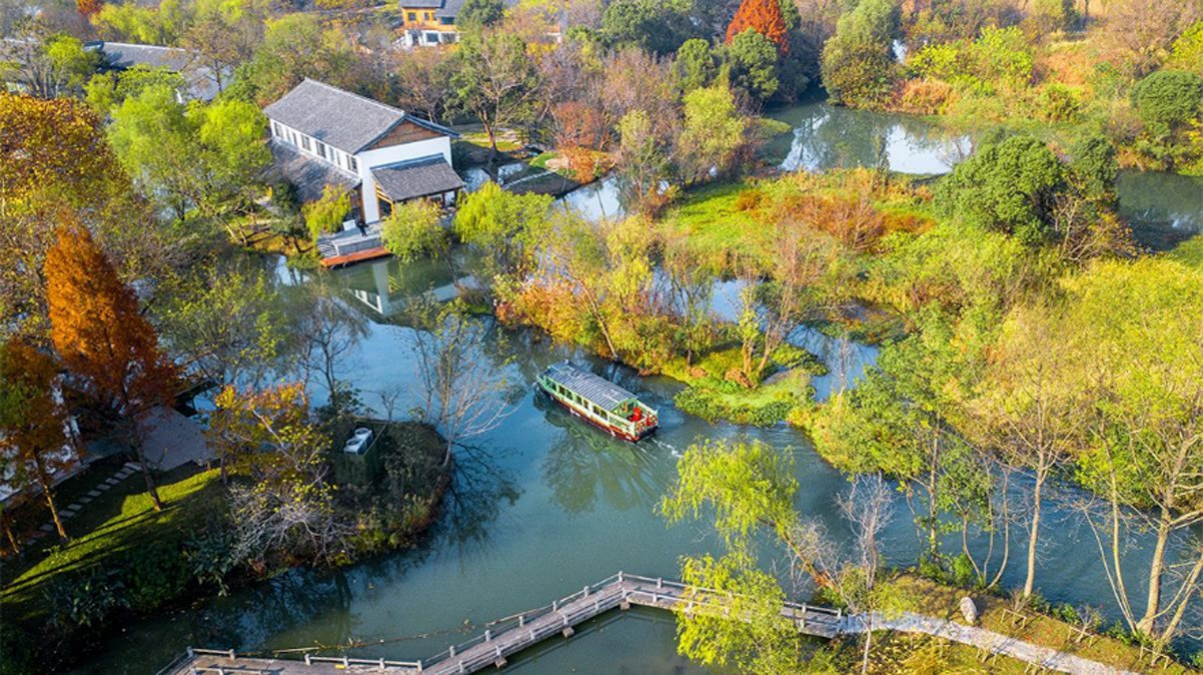
Survey region of Xixi National Wetland Park.

The vast majority of restoration studies often adopted a convenient student sample in a laboratory environment. However, this approach might cause sample and context bias [33]. To avoid the limitations existed in previous studies, we collected data from visitors who are visiting the study site at present (Fig 3). The study was approved by the ethics committee of Nanjing Forestry University (NJFU Ref No. 2019/1012). The questionnaires were first distributed from October 21–25, 2019, which is the peak season of Xixi National Wetland Park. Questionnaires were issued only after we obtained the verbal agreement from the visitors. Before issuing the questionnaire, visitors were asked whether they agree to participate this survey anonymously, if they visited most of the scenic spots in the Xixi Wetland National Park, and whether they are adults. Overall, 600 visitors were selected randomly and 562 valid questionnaires were extracted for data analysis.

Since the COVID-19 crisis, Xixi National Wetland Park enforced stringent control measures as the park did not open completely until September 2020. The second on-site survey was conducted on China’s National Day holiday (October 6–8, 2020). We randomly selected 500 domestic visitors to participate in the survey, and finally collected 341 valid questionnaires. All the participants orally agreed to take part in the questionnaire.

There are 562 samples in the pre-COVID-19 group, 51.42% were female. Their average age was 39.23 years (standard deviation [SD] = 7.36) and 59.79% of the samples possess a diploma and above degree. For the samples in the post-COVID-19 group, 61.29% were female. Their average age was 38.17 years (SD = 6.52) and 77.13% of the samples possess a diploma and above degree. The differences in demographic characteristics were not significant, and thus the two groups of data are suitable for comparison.

### Analytical strategies

Descriptive statistics and internal consistencies were analyzed through SPSS 27.0. The research assumptions of the conceptual model were examined by the Partial Least Squares structural equation modelling (PLS-SEM), and the software Smart PLS 3.20. The relationships among being away, compatibility, extent, and fascination, before and after the COVID-19 pandemic is less, compared to previous studies. It means this subject is at the stage of exploration. PLS-SEM is good at predicting instead of confirming the key constructs as it explains the residual variance of the latent variables [34]. Given that this study aims at the evaluation of a set of predictive relationships among various restorative components, the PLS-SME approach is considered more appropriate than the Covariance-based structural equation modelling (CB-SEM).

## Results

### Descriptive analysis

The mean value of all items was higher than 3.500, both in the two groups. For the pre-COVID-19 samples, the mean value of being away was the highest among all the restorative components, followed by fascination, compatibility, and extent. For the post-COVID-19 samples, however, being away had the lowest mean value, followed by compatibility, extent, and fascination. The perceived stress level of the post-COVID-19 group was higher than the pre-COVID-19 counterparties.

### Common method variance

When the questionnaires are distributed at the same time to similar respondents in a self-reported survey, Common Method Variance (CMV) would be the major concern. CMV refers to the presence of a spurious correlation between two variables caused by a common third variable when they are measured by the same method [35]. As recommended by Podsakoff, Harman’s single-factor test was used to examine the potential of CMV in this study [36]. As a result, only 22.65% of the total variance was explained by the first factor, revealing that this study was not pervasively affected by CMV.

### Measurement model

The construct of Being-away (BA) are posited as second-order construct that is comprised of two dimensions: novelty and escape. We first compared the first- and second-order confirmatory factor analysis (CFA) by the target coefficient. The results indicated that the target coefficients of novelty is 0.769 while the target coefficients of escape is 0.863. It means that 76.9% to 86.3% of the variation among the first-order constructs can be explained by the second-order CFA. Therefore, the data had a better fit index.

As indicated in Table 1, the measurement model contained four constructs: being away, compatibility, extent, and fascination. The loading of each construct on its associated latent variable (LV) was higher than 0.700, suggesting that the reliability of each indicator was acceptable [37]. Moreover, the composite reliability (CR) of all reflective LVs was higher than 0.800, exceeding the threshold of 0.700 [38], indicating that the measurement model were reliability. The average variance extracted (AVE) revealed the convergent validity of each LV. For each group, all the AVE values were higher than 0.500 [39], supporting the validity of the model.

**Table 1:** Individual item reliability and construct validity.

Constructs/items	loading		CR		AVE	
	Pre-COVID-19	Post-COVID-19	Pre-COVID-19	Post-COVID-19	Pre-COVID-19	Post-COVID-19
**Fascination**			0.841	0.917	0.578	0.744
FA1	0.762	0.864				
FA2	0.863	0.888				
FA3	0.833	0.876				
FA4	0.805	0.810				
**Being-away**			0.882	0.911	0.727	0.729
Novelty1	0.822	0.833				
Novelty2	0.854	0.889				
Escape1	0.878	0.856				
Escape2	0.861	0.841				
**Extent**			0.862	0.930	0.596	0.767
EX1	0.828	0.867				
EX2	0.832	0.901				
EX3	0.781	0.889				
EX4	0.762	0.856				
**Compatibility**			0.857	0.879	0.622	0.643
CP1	0.871	0.778				
CP2	0.884	0.752				
CP3	0.762	0.840				
CP4	0.831	0.833				

The PLS-SEM tends to overestimate indicator loadings. Therefore, the heterotrait-monotrait ratio of correlations (HTMT) is required to evaluate the discriminant validity [38]. Discriminant validity describes to what extent each LV is distinct from other constructs in the measurement model [40]. The Fornell–Larcker criterion suggests that the conservative criterion of HTMT should be lower than 0.85 [41]. For each group-specific model estimation, the highest score of all the conservative criteria for HTMT was 0.682, revealing that the discriminant validity was established. Together, the examination of reliability and validity lend sufficient confidence that the measurement had a good fitness.

### Structural model

To avoid model misspecification, Heseler et al. introduced the Standardized Root Mean Square Residual (SRMR), Unweighted Least Squares Discrepancy (d_ULS_) and geodesic discrepancy (d_G_) as a goodness-of-fit measure for PLS-SEM [34]. Before proceeding to test the structural model, we tested the model fit. The data and measurement model is suitable if the value of SRMR is lower than 0.080 and all the discrepancies are below the 95% quantile of bootstrap discrepancies (HI_95_). The SRMR value was 0.028 as well as both the d_ULS_ and the d_G_ were lower than bootstrap HI_95_. These results suggest very good measurement model fit. Overall, the data are coherent with the combination of factors and composites in the measurement model.

**Fig 4:**
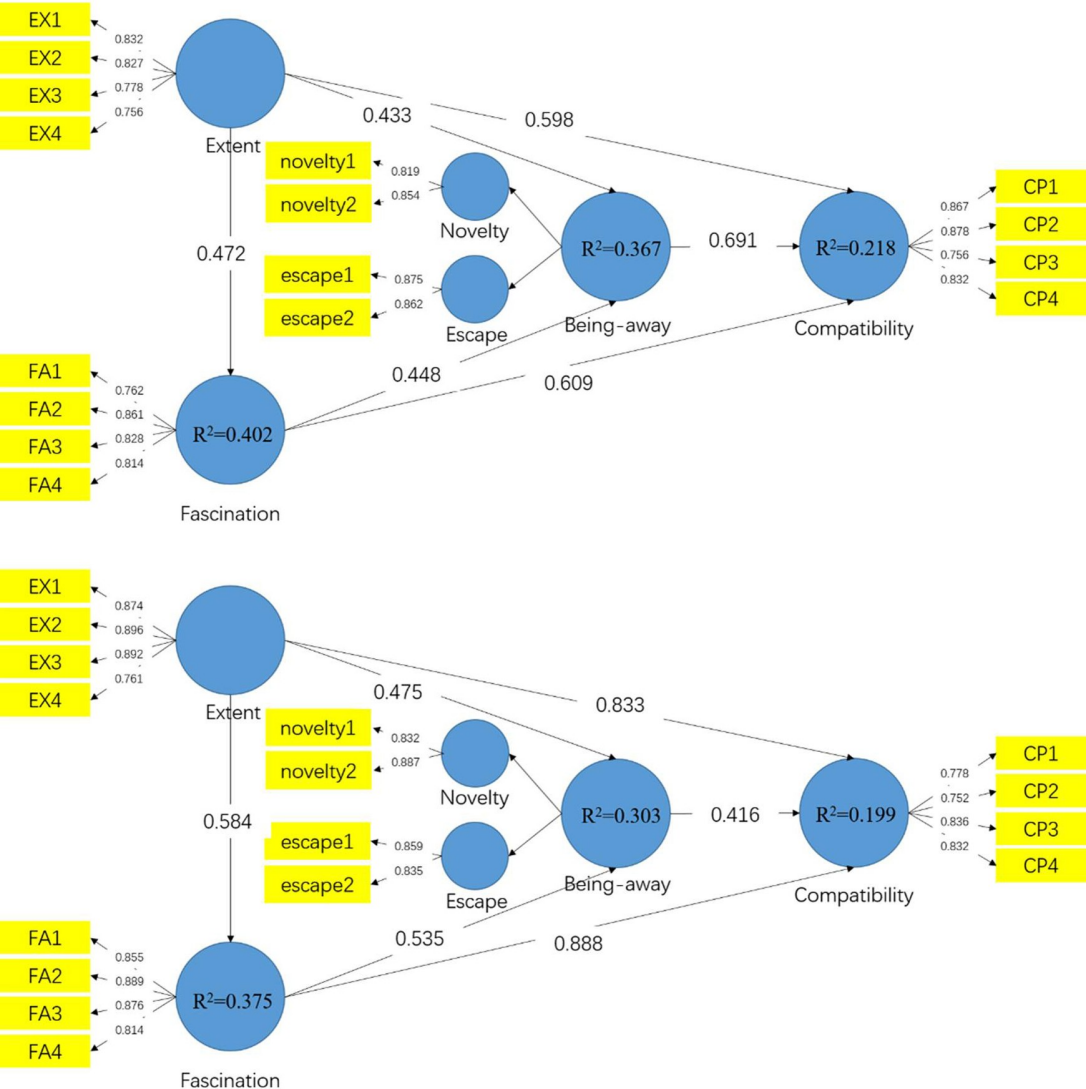
**a**. Results of the structural model for the pre-COVID-19 group. **b**. Results of the structural model for the post-COVID-19 group.

R^2^ indicates the extent to which variables explain the variance [42]. The R^2^ in both groups exceed the threshold of 0.100 (Fig 4A and 4B), suggesting that the structural model has an appropriate predictive power. Moreover, the Q^2^ values of all the endogenous constructs are well above zero, indicating the predictive relevance of the model [43]. All the hypotheses are evaluated through a bootstrapping procedure. Fig 4 shows the PLS results, which indicate that fascination is significantly influenced by extent (β_pre_ = 0.472, β_post_ = 0.584, P<0.001), being away is significantly influenced by extent (β_pre_ = 0.433, β_post_ = 0.475, P<0.001), and compatibility is also significant influenced by extent (βpre = 0.598, βpost = 0.833, P<0.001); fascination direct affect being away (β_pre_ = 0.448, β_post_ = 0.535, P<0.001) as well as compatibility (β_pre_ = 0.609, β_pos_t = 0.888, P<0.001); being away has significant effects on compatibility (β_pre_ = 0.691, β_post_ = 0.416, P<0.001). Thus, H1a, H1b, H1c, H1d, H1f and H1g are all supported in both groups.

To establish the mediating effects, the Z statistic was applied to test if the Soble Z value is higher than 1.96 (p<0.050) [44]. As shown in Table 2, the mediating effects of being away in the post-COVID-19 group are not supported since the Soble Z values do not exceed the threshold of 1.960. Besides, the mediating effect of fascination in both groups and the mediating effect of being away in the pre-COVID group are supported. We further applied the variance accounted for (VAF) value to estimate the ratio of the indirect effect on total effect. As Hair et al. suggested, a VAF less than 20% represents no mediation, 20% to 80% represents partial mediation, and larger than 80% represents full mediation [38].

**Table 2:** The examination of mediating effects.

	Extent->fascination->being away		Extent->fascination->compatibility		extent->being away->compatibility		fascination->being away->compatibility	
	pre-COVID-19	post-COVID-19	pre-COVID-19	post-COVID-19	pre-COVID-19	post-COVID-19	pre-COVID-19	post-COVID-19
Indirect effects	0.213	0.314	0.287	0.513	0.302	0.192	0.314	0.208
Total effects	0.644	0.788	0.891	1.342	0.904	1.022	1.017	1.103
Sobel z test	7.283	6.252	11.238	9.945	18.530	0.861	9.739	1.126
VAF	32.815%	39.242%	32.581%	38.056%	33.334%	18.632%	33.698%	19.087%
Support	Partial mediation	Partial mediation	Partial mediation	Partial mediation	Partial mediation	No mediation	Partial mediation	No mediation

Table 2 also concludes that for both groups, fascination played a partial mediating effect between extent and being away (VAF_pre_ = 32.815%, VAF_post_ = 39.242%) as well as between extent and compatibility (VAF_pre_ = 32.581%, VAF_post_ = 38.056%). Therefore, H2a and H2b are supported. Being-away partially mediated the relationship between extent and compatibility (VAF_pre_ = 33.334%, VAF_post_ = 18.632%) as well as between fascination and compatibility (VAF_pre_ = 33.698%, VAF_post_ = 19.087%) only in the pre-COVID-19 group. Therefore H3a and H3b are supported in the pre-COVID-19 group.

Conceptually, the categorical moderator variable compares the group-specific effects and thus the moderating effects could be regarded as a special case of multi-group analysis (MGA) [45]. The perceived stress level separate the visitors before and after the COVID-19 pandemic into two group: the pre-COVID-19 group with low stress level and the post-COVID-19 group with high stress level. In this regard, the MGA was utilized to test whether the perceived stress level induced by COVID-19 moderates the relationship between the four components of the restorative soundscape.

Before performing a MGA to compare the path coefficients between the two groups, the established primary concern is whether the construct measures are invariant across each group [46]. Hair suggested the measurement invariance of the composite method (MICOM) to test the measurement invariance in PLS-SEM [37]. MICOM involves configural invariance assessment, compositional invariance assessment, as well as an equal means and variances assessment. The results show that the three types of invariances all fall within the confidence interval. Therefore, a MGA is possible.

Partial least squares-based multi-group analysis (PLS-MGA) was performed to test the moderating effects of PSS. This method is useful to examine differences across different categories in the case of categorical data [45]. The Henseler’s bootstrap-based MGA and the permutation test were conducted to compare the path coefficients between the two groups. There are significant differences between specific path coefficients when a p-value of path coefficients differences is lower than 0.05 or higher than 0.95 at the 5% level [39]. Table 3 shows that the pre- and post-COVID-19 groups are significantly different in relation between extent and fascination (β_pre_ = 0.472, β_post_ = 0.584, p = 0.030**), extent and compatibility (β_pre_ = 0.598, β_post_ = 0.833, p = 0.000***), as well as fascination and compatibility (β_pre_ = 0.609, β_post_ = 0.888, p = 0.000***). In addition, there are significant differences for the path coefficients from being away to compatibility (β_pre_ = 0.691, β_post_ = 0.416, p = 0.000***). Therefore, moderating effects of the perceived stress level on the relation between extent, fascination, being away, and compatibility, as well as extent and fascination, were examined through PLS-SEM bootstrapping technique. However, the results of MGA indicates that the perceived stress level does not have moderating effects between extent and being away (β_pre_ = 0.433, β_post_ = 0.475, p = 0.114) as well as fascination and being away (β_pre_ = 0.448, β_post_ = 0.535, p = 0.107) across both groups.

**Table 3:** Results of MGA.

Relationships	Path Coefficients	Path Coefficients	CIs	CIs	Path coefficient differences	P-value	P-value	Supported
			(Bias corrected)	(Bias corrected)		Henseler’s MGA	Permutation test	
	pre-COVID-19	post-COVID-19	pre- COVID-19	post- COVID-19				
extent->fascination	0.472	0.584	[0.230, 0.571]	[0.351, 0.682]	0.112	0.030**	0.021**	H1a √
extent->being away	0.433	0.475	[0.367, 0.701]	[0.321, 0.533]	0.042	0.114	0.260	H1b ×
extent->compatibility	0.598	0.833	[0.452, 0.689]	[0.766, 0.867]	0.235	0.000***	0.000***	H1c √
fascination->being away	0.448	0.535	[0.315, 0.514]	[0.522, 0.612]	0.087	0.107	0.171	H1d ×
fascination->compatibility	0.609	0.888	[0.445, 0.695]	[0.671, 0.960]	0.279	0.000***	0.000***	H1f √
being away->compatibility	0.691	0.416	[0.631, 0.862]	[0.367, 0.533]	-0.275	0.000***	0.000***	H1g √

## Discussion

Few research studies on restoration explored the factors of natural soundscapes that restore people’s direct attention fatigue. To fill this gap in literature, this study developed and validated a conceptual model to describe the restorative process of these components in improving the mental health of pre- and post-COVID-19 visitors in a nature reserve.

The scores of all variables from pre- and post-COVID-19 groups indicate the restorative benefits of natural soundscapes in Xixi National Wetland Park. Natural soundscapes are more novel than visualscapes in the natural environment to attract visitors and restore their directed attentional fatigue [47]. Accordingly, visitors could define the meaning of soundscape as being in harmony with their natural surroundings, leading to mental restoration. The results of this study challenges the previous argument that the restorative effects of nature are dominated by visual elements [13]. Moreover, this study shed light on the inter relationship among the restorative components (Fig 4), which are on a different hierarchical level: extent and fascination describe the characteristics of natural soundscape, being away refers to the individual’s physical or psychological statue, and compatibility is influenced by the three previously mentioned components [48, 49]. Specifically, when natural soundscapes have explorative potential (extent) and attention-holding properties (fascination), they induce a novel situation quite different from daily routine (being away), and thus promote a coherence between the individual and the natural soundscapes (compatibility).

The results of MGA showed that the relationships of the restorative components are quite different between the pre- and post-COVID-19 groups (Table 3). The variation in path coefficients are related to the moderating effects of perceived stress level and thus is relevant to the context [14]. For the post-COVID-19 group, the effects of extent on fascination, and the effects of extent and fascination on compatibility were larger when compared with the pre-COVID-19 group. The COVID-19 pandemic is a significant stressor for mental health. It has profoundly altered people’s daily lives and created multiple societal challenges [50]. The post-COVID-19 group with high stress levels is more sensitive when listening to natural soundscapes, leading to larger effects of extent and fascination on compatibility. This finding is in agreement with the study of Grahn and Stigsdotter that the higher the perceived stress level, the stronger the restorativeness of natural elements [51] Furthermore, the extreme action of ban on tourism reduced the environmental disruption and promoted the ecological resilience. The natural soundscapes increased post COVID-19. Contact with various natural soundscapes offers individuals more opportunities to reorganize the connectedness of humans with nature, and uplift their mental state [52].

Interestingly, the influence of being away on compatibility was smaller while the mediation role of being away was non-significant for the post-COVID-19 samples. This is inconsistent with previous studies [53]. The threat of infection due to the COVID-19 pandemic was everywhere and there was nowhere to run [1]; the strict isolation measures confined people to their homes [54]. As a result, the willingness of physical or psychological departure from the familiar situation became weaker for the post-COVID-19 samples and the effects of being away on other restorative components were less.

From a practical angle, the development of the conceptual model to describe the relation between natural soundscape components and mental health can also be employed by practitioners as an instrument to create more profit. Compared with visualscape, smellscape, and tactilescape, natural soundscapes help visitors to maintain a proper social distance during the COVID-19 pandemic, which is critical in the current public health emergency [14]. Environmental regulatory policies should pay attention to the protection of natural soundscapes in destination management. It is also important for government managers to be aware that noise reduction is not an effective method for health promotion; the sustainable development of natural soundscapes to conduct a pleasant sonic environment is more important to the public mental health, especially during the COVID-19 pandemic.

In addition, this study can be helpful for DMOs in improving the effectiveness of auditory-based promotional efforts. This study identifies the restorative components of natural soundscape that can best foster visitor recovery from attention fatigue. For example, visitors prefer natural soundscapes that are not far away from their daily life during the post-COVID-19 period. They are reluctant to go far from their familiar environment. Besides, the disordered soundscapes also limit visitors to explore the large scope of nature while requiring more attention to resolve the confusion, which is incompatible with the visitors’ demands of renewal and recovery. Therefore, one strategy for DMOs is to pay attention to natural soundscapes during short vacations, protect and design the natural soundscapes with clearly organized, and promote them to be harmonious with their surroundings. This way, visitors could in a pleasant sonic environment and experience full mental restoration by eliminating the fear, anxiety, sadness and loneliness caused by the outbreak of COVID-19.

## Conclusions

This study conducted a comparison of mental restoration before and after the COVID-19 pandemic in Xixi National Wetland Park to explore the mechanism of natural soundscapes for mental restoration. Results show that natural soundscapes have great restorative benefits for visitors, and the inter relationships of the restorative components are significantly different when influenced by the great threaten of COVID-19 pandemic. Especially for visitors in the post-COVID period, the effects of extent and fascination on compatibility are stronger and more significant, while the being away is less supportive on compatibility.

Furthermore, this paper also provide practical implications for policy makers and DMOs to develop a wellness product in the current global health crisis. Since the differences of the perceived restorativeness soundscape between pre- and post- COVID-19 sample, government managers should strengthen environmental regulation and perfect the environmental regulation system to protect the sustainable development of natural soundscape after COVID-19 outbreak. Creating opportunities for auditory interaction with the destination can contribute to enhance public mental health as well as eliminate the COVID-19 crisis. DMOs should also be cognizant the importance of natural soundscape since a favorable assessment of the soundscape characteristics restores visitors’ mental fatigue as well as strengthens their loyalty. A rational utilization of the restorative components of natural soundscape will be promoted in post-COVID-19 period.

A number of limitations of this study must be acknowledged. The analysis and results are limited to the cross-sectional data before and after the outbreak of COVID-19 [55]. Diachronic studies with long-term survey data is necessary to understand the restorative capacity of natural soundscapes when the COVID-19 pandemic is well over. Additionally, the results originated from self-report scales lack comparison with physiological data from visitors, and the psychophysiological measurement is scarce to test the visitors’ mental state. Future research would combine mixed methodologies to enhance the reliability and validity of the results [56]. Finally, the present study only considers natural soundscapes as a whole. In the future, we will compare different types of soundscapes in various environments to examine their restorative effects. With this comparison, the findings of case studies could be translated into generalized statements of universal significance.

## Supporting information

S1 Data (XLSX)
